# Low Levels of Knowledge, Attitudes and Preventive Practices on Leptospirosis among a Rural Community in Hulu Langat District, Selangor, Malaysia

**DOI:** 10.3390/ijerph15040693

**Published:** 2018-04-06

**Authors:** Noramira Nozmi, Suhailah Samsudin, Surianti Sukeri, Mohd Nazri Shafei, Wan Mohd Zahiruddin Wan Mohd, Zawaha Idris, Wan Nor Arifin, Norazlin Idris, Siti Nor Sakinah Saudi, Nurul Munirah Abdullah, Zainudin Abdul Wahab, Tengku Zetty Maztura Tengku Jamaluddin, Hejar Abd Rahman, Siti Norbaya Masri, Aziah Daud, Malina Osman, Rukman Awang Hamat

**Affiliations:** 1 Department of Medical Microbiology and Parasitology, Faculty of Medicine and Health Sciences, Universiti Putra Malaysia, Serdang, Selangor 43400, Malaysia; miranozmi@live.com (N.N.); suhailah.sam@gmail.com (S.S.); sakinahsaudi@gmail.com (S.N.S.S.); nurulmunirahabdullah@gmail.com (N.M.A.); tengkuzetty@upm.edu.my (T.Z.M.T.J.); sitinorbaya@upm.edu.my (S.N.M.); malinaosman@upm.edu.my (M.O.); 2 Department of Community Medicine, School of Medical Sciences, Universiti Sains Malaysia, Kubang Kerian, Kelantan 16150, Malaysia; surianti@usm.my (S.S.); drnazri@usm.my (M.N.S.); drzahir@usm.my (W.M.Z.W.M.); norazlin@usm.my (N.I.); aziahkb@usm.my (A.D.); 3 Health Promotion Unit, Penang State Health Department, Floor 7, Bangunan Persekutuan, Jalan Anson, Penang 10400, Malaysia; hjhzawaha@yahoo.com; 4 Unit of Biostatistics and Research Methodology, School of Medical Sciences, Universiti Sains Malaysia, Kubang Kerian, Kelantan 16150, Malaysia; wnarifin@usm.my; 5 Health Department of Federal Territory Kuala Lumpur & Putrajaya, Jalan Cenderasari, Kuala Lumpur 50590, Malaysia; zwah58@gmail.com; 6 Department of Community Health, Faculty of Medicine and Health Sciences, Universiti Putra Malaysia, Serdang, Selangor 43400, Malaysia; hejar@upm.edu.my

**Keywords:** knowledge, attitude, preventive practice, leptospirosis, rural community, water bodies, rodents

## Abstract

Little is known on the knowledge, attitudes and preventive practices (KAP) of leptospirosis worldwide. This study embarked on assessing the KAP of leptospirosis among rural communities in Malaysia. A total of 444 participants (223 male; 221 female) aged between 18 and 81 years old were surveyed by using a self-administered questionnaire. A majority of participants had poor knowledge level (57.0%), unacceptable attitudes (90.3%) and unacceptable preventive practices (69.1%) on leptospirosis, and only 29.7% knew “rat-urine disease” as leptospirosis. Only 34.2% of the participants knew the bacteria could enter via wound lesions. Ethnicity and income were strongly associated with knowledge level and preventive practices, respectively (*p*-values < 0.05). As for attitudes, ethnicity, income and education type were significantly associated (*p*-values < 0.05). Only 36.5% of the participants were willing to see a doctor and did not mind if their house or surrounding area is dirty (59.7%). Surprisingly, only 32.9% had used rubber boots during floods. By logistic regression analysis, ethnicity was the only significant predictor for both knowledge level (an odds ratio (AOR) = 0.39, 95% confidence interval (CI) = 0.222–0.680) and preventive practices (AOR = 1.81, 95% CI = 1.204–2.734). Ethnicity (AOR = 0.40, 95% CI = 0.239–0.665), income (AOR = 1.58, 95% CI = 1.041–2.385) and education type (AOR = 3.69, 95% CI = 1.237–10.986) were strong predictors for attitudes. Among the KAP variables, attitude (AOR = 4.357, 95% CI = 2.613–7.264) was the only predictor for the preventive practices by logistic regression analysis. The KAP elements on leptospirosis are still lacking and poor health seeking behavior and attitudes are of our utmost concern. Thus, effective strategies should be planned to impart knowledge, and develop proactive approaches and good preventive modules on leptospirosis to this leptospirosis-prone community.

## 1. Introduction

Leptospirosis—a neglected zoonotic disease caused by pathogenic *Leptospira* strains—is now becoming a global threat for causing significant morbidity and mortality in humans in both rural and urban settings worldwide [1,2,3,4,5]. Recently, more than 300 serovars and 20 species of these pathogenic strains have been identified and mammals, including rodents, are among the important reservoirs known to harbor these spirochetes [6,7,8]. Human leptospirosis is acquired through direct or indirect contact with water or soil contaminated by the urine, blood or tissues of infected mammals [3,9]. The disease has a wide clinical spectrum ranging from asymptomatic to hemorrhagic phenomenon and even death [2]. Nonetheless, the clinical manifestations of human leptospirosis possess a significant challenge to clinicians in managing the disease, since other tropical diseases such as dengue and malaria rather share similar symptomatology and epidemiology, albeit the pathogenesis pathways are different [10,11].

Globally, leptospirosis is considered as a re-emerging infectious disease and more than one million cases have been reported every year with the estimated incidence of 0.1 to 975 cases per 100,000 population annually [1,12]. The disease burden is largely centered in tropical and subtropical regions such as Southeast Asia and also in Central and South America regions [13,14,15,16]. Several contributing factors have been highlighted for high morbidity and mortality rates in these regions. The geographical location with frequent rainfall and floods, climate change, high number of animal reservoirs, human activities and other socio-epidemiological influences are attributable to the high incidence of leptospirosis [13,17,18,19,20].

Several outbreaks have been reported following climate change and human activities in Malaysia. For instance, an Eco-Challenge held in Borneo Island has recorded 29 of 304 athletes from 26 countries contracted leptospirosis [21]. Another outbreak has been reported involving 46 boys and young adults aged 8 to 19 years old who swam in a creek in Sabah with one succumbed to leptospirosis [22]. As for the natural disasters, 20 human leptospirosis cases with two deaths were reported following the prolonged flood during monsoon season that hit Johor, Malaysia [23]. Recently, a few north-eastern states of Malaysia such as Kelantan and Selangor recorded a high number of human leptospirosis cases in 2014 due to flood as well [24].

With the wealth of scientific data, our understanding of leptospirosis in Malaysia seems to be significantly improved. However, in order to ensure good control and preventive measures, surveys of knowledge, attitude, and preventive practices (KAP) among the public are urgently needed. Information from these surveys could also provide strategies to improve or evaluate existing programs as well as to identify potential interventions for behavioral changes. It is known that knowledge levels and perceptions are important key drivers for positive behavior [25]. To date, data on KAP studies on leptospirosis among rural population are still lacking in Malaysia. Herein, we present findings obtained from our rural communities to determine the socio-demographic characteristics, levels of knowledge, attitude, and preventive practices of leptospirosis.

## 2. Materials and Methods

### 2.1. Sample

The study commenced in Hulu Langat District, Selangor from February 2016 and ended in June 2017. The population of Hulu Langat District is approximately 1,156,585 people [26]. Hulu Langat has three waterfalls which are Air Terjun Sungai Gabai, Air Terjun Sungai Lepoh, and Air Terjun Perdik. It also has six rivers which are Sungai Congkak, Sungai Tekali, Sungai Tekala, Sungai Langat, Sungai Lui, and Sungai Pangsun. This district has attracted many visitors from Selangor and other states of Malaysia for its majestic waterfalls and shallow rivers. Recently, it has been declared as one of the hotspot areas for leptospirosis [24]. Leptospirosis cases were recorded to be the highest among other states in 2015 (2610 cases) based on the data obtained from Selangor State Health Department Office. In addition, 48% of the total deaths due to leptospirosis in Selangor were reported in Hulu Langat. Approval to conduct the study was obtained from the Faculty of Medicine and Health Sciences Ethical Review Committee Board, Universiti Putra Malaysia (UPM/TNCPI/RMC/JKEUPM/1.4.18.2).

### 2.2. Study Design

The study utilized a cross-sectional study design.

### 2.3. Study Population

Selangor is divided by nine districts and Hulu Langat has 19 villages within its territory. The study locations (4 villages) were selected by simple random sampling using a computer random generator to avoid any potential biases. The list of the villages was obtained from Hulu Langat District and Land Office. The aim of the study was briefed to each individual occupant residing in the villages in the rural area. Those who did not fulfill the criteria such as non-permanent resident and aged less than 18 years old were excluded in the study. Those who were eligible and voluntarily agreed to participate were asked to give their written consent. Each participant was approached by the investigators to complete a set of questionnaires using hard copies. The questionnaires were designed in Bahasa Malaysia (a local dialect), labelled and kept in a sealed envelope to ensure the confidentiality.

### 2.4. Instrument

The study used a validated KAP questionnaire on leptospirosis in Malaysia which has been adopted and modified accordingly [27]. The content validity of the study instrument was performed by cross-checking and authentication from experts in the field of study. The questionnaire was pre-tested among 30 Hulu Langat rural residents before the commencement of the study. Cronbach’s Alpha coefficient with acceptable results were obtained (knowledge, α = 0.710; attitude, α = 0.837; and practices, α = 0.956). Collected data were obtained based on 4 categories: (1) socio-demographic characteristics, (2) source of information and (3) variables of knowledge, attitude, and preventive practices of leptospirosis. Rural community was defined as residents who live outside urban areas including settlements with a population less than 10,000 residents, agricultural areas, forest and water bodies [26]. The divorced individual was considered unmarried. Informal education was defined as an educational model that comes spontaneously during the learning process and does not necessarily have a pre-defined curriculum [28].

Awareness of the participants on leptospirosis was assessed if the study participants had heard of the “rat-urine disease” as well as the source of the information. The terminology of “rat-urine disease” was adopted as it is commonly used by the local media to represent leptospirosis. The participants were asked on 8 items about the causative agent, how leptospirosis could be diagnosed, how leptospirosis could be transmitted, how leptospirosis could be manifested in man and its complications, and preventive measures that could be taken for instance, avoid wading through flood and swimming in contaminated river, lake and others. Participants were given 3 options such as “Correct”, “Wrong” and “Do not know”. Each correct answer was given as 1, while a 0 score was given to wrong answer and do not know. Participants with the knowledge scores of 60% and above were considered as having good knowledge, while those who scored below 60% were categorized as having poor knowledge [29].

Attitudes related to leptospirosis were determined by assessing 16 items related to environmental exposures to leptospirosis and perception on wearing protective equipment as well as towards readiness of wearing protective equipment, avoidance of exposure, participation in control and prevention activities, and health seeking behaviors. The responses were gathered using a 5-point Likert scale where 5, 4, 3, 2 and 1 corresponded to strongly agree, agree, maybe, disagree and strongly disagree, respectively. As for the negative statements, the scales were reversely arranged and recorded. Those participants with attitude scores of 80% and above were categorized as having acceptable attitude, while those who scored below 80% were considered as having unacceptable attitude [30].

The preventive practice questions contained 17 items (ensuring no rats around their houses, avoidance of risky activities and behaviors, rubbish and waste management, hand hygiene practices, food hygiene practices, treatment obtained, and opting for clean restaurant). Participants were asked to give a response of “Not applicable”, “Never”, “Sometimes”, “Most of the time”, and “All of the time” and scored as “0”, “1”, “2”, “3” and “4”, respectively. Those participants that had scores 80% and above were categorized as having acceptable preventive practice, while those with scores lower than 80% were categorized as having unacceptable preventive practice [31].

### 2.5. Data Analysis

SPSS Version 23 statistical software (SPSS Inc., Chicago, IL, USA) was used for the data analysis. Descriptive statistics were used to determine the socio-demographic factors and the KAP scores. Associations between categorical variables were analyzed by Chi square. In all cases, *p*-value < 0.05 was considered to be statistically significant at 95% CI. As for the significant associations, logistic regression was used for the respective variables.

## 3. Results

### 3.1. Demographic Data

In total, 444 participants were involved in the study. Males and females participated almost equally (50.2% vs. 49.8%). The age of the participants ranged from 18–81 years old with the mean age of 34.1 (±14.6). Malays (83.1%) outnumbered the Chinese (2.9%), Indian (6.8%) and others (7.2%). A majority of the participants were married compared to unmarried (65.3% vs. 34.7%), and almost a quarter (23.8%) had two children. Most of the participants received formal education than non-formal one (96.8% vs. 3.2%). However, 37.4% of the participants were unemployed and 43.5% earned less than 1500 Malaysian ringgit. The socio-demographic characteristics of the participants are shown in the Supplementary Material (Table S1).

### 3.2. KAP on Leptospirosis

#### 3.2.1. Awareness and Source of Information on Leptospirosis

A majority of the participants (87.4%) had heard about “rat-urine disease”. Most of the participants (75.5%) identified the television/radio as the main source of information. Almost half (51.0%) had the information from the newspaper. Only 17.8% and 15.5% obtained the information from the doctor and magazine, respectively. Sources of information are shown in the Supplementary Materials (Table S2).

#### 3.2.2. Knowledge on Leptospirosis

Overall, 43% and 57% of the participants had good and poor knowledge levels, respectively. Only 29.7% of the participants knew “rat-urine disease” is also known as leptospirosis and 69.1% of the participants were not aware of it. For further analysis and discussion, we used leptospirosis to represent “rat-urine disease” which was perceived by the rural communities. Interestingly, 66.7% correctly responded to leptospirosis as a zoonotic disease and 58.6% knew that leptospirosis is caused by bacteria. The participants also knew that leptospirosis could be diagnosed by blood test (61.0%). Meanwhile, contaminated water and food were the most well known as the routes of transmission by 61.9% and 60.4% of the study participants, respectively. Only 34.2% of the participants knew the bacteria could enter the wound and 58.1% were not aware of it. Surprisingly, shaking hands with infected person was thought of as a route of transmission (12.8%). Regarding the signs and symptoms of leptospirosis, 68.0% and 52.5% of the participants knew fever and body ache are symptoms of leptospirosis. However, only 24.5% of the participants knew yellowing of the eyes is the sign of leptospirosis, and the majority of participants (66.4%) were not aware of it. As for the complications, only 39.0% and 36.9% of the participants knew leptospirosis could lead to kidney failure and liver damage, respectively.

Concerning the preventive practices of leptospirosis, a majority of the participants (81.3%) knew leptospirosis could be prevented by ensuring the residential area is free of rubbish, followed by drinking clean water supply (77.7%), practicing good personal hygiene (77.7%), not swimming in contaminated freshwater bodies (75.5%) and not wading through flood (67.1%). However, wearing rubber gloves as a protective measure was known by only 55.2% of the participants. Table 1 shows the participants’ knowledge of leptospirosis.

#### 3.2.3. Attitudes on Leptospirosis

Regarding attitudes towards readiness of wearing protective equipment, only 37.8% and 33.3% of participants were strongly agreed and agreed, respectively, on the willingness of wearing gloves while collecting the rubbish. Half of the participants (50.7%) had strongly agreed to make sure the dustbin is always covered. Less than half of participants had strongly agreed to make sure their family members would participate in cleaning activities (46.2%), and participated in prevention and control activities initiated by the authority (45.0%). Only 36.5% had strongly agreed to see a doctor if the participants have fever during a leptospirosis outbreak. Astonishingly, more than half did not mind if the house or its surrounding area is dirty (59.7%) and did not care if the participants find rats in the house or its surrounding area (55.4%). Table 2 shows the participants’ attitudes related to leptospirosis.

#### 3.2.4. Preventive Practices on Leptospirosis

With regard to cleaning activities as preventive measures 63.5%, 60.6% and 49.3% of the participants had washed their hands with a soap after handling the rubbish or waste, their cooking tools and cleaned the house area from any rubbish or waste, respectively. A small percentage of the participants (6.8%) had gone for a picnic at the contaminated river/lake/waterfall for the past six months. In addition, a majority of the participants (64.0%) had kept the food in a covered container and 61.3% of the participants had covered the dustbin to prevent rats. Less than half (42.6%) had covered the wound or small cut while handling the rubbish or waste. Surprisingly, only 32.9% of the participants had used rubber boots while wading through the flood (Table 3).

### 3.3. KAP Analysis with Other Parameters

The data analysis on knowledge level of leptospirosis showed that Malays had good knowledge level than non-Malays (46.6% vs. 25.3%; prevalence ratio (PR) = 1.840, 95% CI = 1.229–2.755). The association of knowledge level and other variables are shown in the Supplementary Materials (Table S3). A very good attitude was demonstrated among Malays than non-Malays (66.7% vs. 44.0%; prevalence ratio (PR) = 1.515, 95% CI = 1.162–1.976). In terms of income, those who earned RM1500 and more had acceptable attitudes compared to those who earned less than RM1500 (69.4% vs. 58.8%; prevalence ratio (PR) = 0.843, 95% CI = 0.748–0.950). Participants who received formal education had acceptable attitudes than those who received non-formal education (64.0% vs. 31.3%; prevalence ratio (PR) = 1.911, 95% CI = 1.341–2.721). Associations between the participants’ attitude according and their respective variables are shown in Table 4. As for the preventive practices against leptospirosis, those who earned RM1500 and more had higher level of acceptable practices than those who earned less than RM1500 (38.8% vs. 25.9%; prevalence ratio (PR) = 1.211, 95% CI = 1.054–1.391). The level of preventive practices and its socio-demographic variables are shown in the Supplementary Materials (Table S4).

In the logistic regression analysis (Table S5), ethnicity was the only significant predictor for knowledge where Malays had 2.6 times higher odds of having good knowledge compared to non-Malays (AOR = 0.39, 95% CI = 0.222–0.680). Malays also had 2.5 times higher odds of having acceptable attitudes than non-Malays (AOR = 0.40, 95% CI = 0.239–0.665). In addition, income and education type were also significant predictors for attitudes. Those who had an income of RM1500 and more were 1.6 times more likely to have acceptable attitudes than those who had less than RM1500 income (AOR = 1.58, 95% CI = 1.041–2.385). Meanwhile, those who had a formal education were 3.7 times more likely to have acceptable attitudes than non-formal one (AOR = 3.69, 95% CI = 1.237–10.986). Table 5 shows the logistic regression analysis predicting the attitude on leptospirosis. As for the preventive practices, income was the only significant predictor. Those who earned RM1500 and more had 1.8 times higher odds of having acceptable practices than those who earned less than RM1500 (AOR = 1.81, 95% CI = 1.204–2.734). The logistic regression analysis is shown in the Supplementary Material (Table S6).

Table S7 shows the associations between preventive practices and knowledge and attitudes of the rural communities. Participants with good knowledge had acceptable practices than those with poor knowledge (37.2% vs. 26.1%; prevalence ratio (PR) = 0.702, 95% CI = 0.532–0.926). In addition, an acceptable level of practices was observed among participants with acceptable attitudes than those with unacceptable attitudes (41.2% vs. 13.3%; prevalence ratio (PR) = 0.323, 95% CI = 0.214–0.489). However, attitude was the only predictor for the preventive practices of leptospirosis by logistic regression analysis (Table S8). Participants with acceptable attitudes had 4.4 times higher odds of having acceptable level of preventive practices than those who had unacceptable attitudes (AOR = 4.357, 95% CI = 2.613–7.264).

## 4. Discussion

Several sero-epidemiological studies have documented the possible association of leptospirosis with certain high risk occupations such as town service workers, soldiers, farmers, food-handlers and people who were involved in recreational activities [20,30,32,33,34]. However, studies to explore the knowledge, attitudes and preventive practices are still lacking. With an increasing trend of number of deaths due to leptospirosis reported annually, an urgent need to gather important data for effective control and preventive plans is imminent.

### 4.1. Knowledge of Leptospirosis on Etiological Agents, Modes of Transmission, Clinical Symptoms, Preventive Measures and Its Complications

As expected, a majority of the members in a rural community in the present study have heard of “rat-urine disease” (87.4%) and a high proportion of the study participants obtained the information from the television and radio (75.5%). However, almost 70% of our rural communities were unaware of the term leptospirosis. In contrast, 90% of urban slum dwellers were aware of leptospirosis [35]. In addition, a higher percentage (87.3%) of rural workers who had heard of leptospirosis by broadcast media were observed in The Philippines [31], but lower (57.0%) among farmers in Ubon Ratchathani province, Thailand [36]. The perception of the disease as “rat-urine disease” has been accepted by our community for many decades as the term was portrayed by Malaysian headlines and broadcast media in several outbreaks or cases involving campers, rescuers and armies, which could be sensational news [37,38]. However, since leptospirosis is not only caused by infected rats but also other mammals [39], accurate information is needed for better understanding of the disease so that effective preventive strategies can be implemented. Surprisingly, only 17.8% of the participants had heard it from doctors in the present study. This is in contrast with a study in Thailand where 94.2% of rural farmers obtained the information from hospital staff [36]. This could be explained by the high endemicity of leptospirosis in the studied area, thus, massive health campaigns could have been initiated for the farmers.

In general, a higher percentage of our rural communities had poor knowledge of leptospirosis than good knowledge (57.0% vs. 43.0%) with the mean knowledge score of 52.1 (SD ± 26.4). Interestingly, higher percentages of people had poor knowledge levels were reported in other studies locally and abroad. For instance, 98.0% and 87.2% of healthy individuals and town service workers had poor knowledge level, respectively [40,41]. However, the cut-off levels for the good knowledge were higher than the present study which could influence the percentages of poor knowledge levels in their studies. In Nigeria, 95.8% of abattoir workers had a poor knowledge level on leptospirosis [42]. Meanwhile, 80.0% of rural farmers in Buriram Province, Thailand had a poor level of knowledge [43]. However, this could be explained by a high percentage of villagers who only received primary school education and lower (73.0%) in their study. Additionally, 58.6% of our rural communities knew leptospirosis is caused by bacteria, and 66.6% of the participants were correctly identified leptospirosis as a zoonotic disease. Higher percentages of people’s understanding of the bacteria as the causative agents were reported in Thailand (77.9%) and The Philippines (91.0%) [36,44]. It seems that the level of knowledge varies according to the socio-demographic profile, geographical location and the type of methodology used.

Based on the data gathered from the questions on the transmission, 70%, 69.8% and 58.1% of members among our rural community were unaware of nose, eye and wounds, respectively as the entry points for *Leptospira*. This is very crucial as these people are living near to the freshwater bodies, and the tendency to get leptospirosis could possibly be high if the study participants are involved in any aquatic activities especially in areas where the environmental sanitation is poor [45]. In contrast, residents in The Philippines knew skin could be the portal of entry (85.8%) [46], and recent findings of rural people residing in 14 cities of The Philippines documented that leptospirosis could enter humans via skin abrasions (71.0%) and mucous membranes (57.0%) [44]. Sakinah et al. [40] reported 66.2% of healthy residents knew *Leptospira* could enter human body via skin cuts. Contaminated water/drinks could only be recognized by our rural communities in the present study as a medium for leptospirosis to enter human body (61.9%). In Brazil, however, only 31.1% of urban slum dwellers knew leptospirosis could be acquired this way [35]. In highly endemic areas in Thailand, contaminated water was regarded as the mode of acquisition by 97.1% of rural villagers [36]. Meanwhile, 57.9% and 25.4% of Sri Lankans who had been involved in leptospirosis outbreak could only recognize contaminated water and drinks, respectively as the modes of acquiring leptospirosis [47]. In the present study, 60.4% of rural villagers knew leptospirosis can be transmitted by contaminated food. Information on contaminated food as a potential source of leptospirosis is lacking in the literature. Only two local studies have documented among healthy population (75.4%) and town service workers (78.9%) [40,41]. It is worth noting that contaminated drinks and food are among the risk factors for leptospirosis [48]. Surprisingly, 62% and 25.5% of our rural communities did not know and wrongly answered, respectively to the question on shaking hands with infected person could transmit leptospirosis. In view of the lack of knowledge of some aspects of the entry points of leptospirosis in the present study, educational programs should be enhanced and specifically oriented to this rural community for the successful prevention of this deadly disease as reported in many reports [49,50].

As for the clinical manifestations of leptospirosis, a majority of our rural communities knew fever (68.0%) and body ache (52.5%) were the symptoms. Higher percentages of fever as the clinical symptom were observed in two studies in Sri langka (93.4% and 86.0%) [29,47]. However, similar finding was observed for the body ache in a study in Sri Langka (53.6%) [29]. Rural communities in the present study were not aware of jaundice as the clinical sign of leptospirosis (66.4%). This is in accordance with poor knowledge of liver damage as a complication of leptospirosis (36.9%) in the present study. Ironically, 14.6% of people who had experienced an outbreak of leptospirosis were still ignorance of jaundice as one of the clinical signs in Sri Langka [47]. Jaundice is a sign observed in patients with leptospirosis, and it is due the accumulation of bilirubin in the blood stream as a result of hepatocellular damage and hepatocyte apoptosis [51]. Death (80.4%) was highly regarded as the main complication, followed by difficulty in breathing (45.3%) in the present study. In contrast, 4.2% of urban slum dwellers in Brazil knew leptospirosis is the deadly disease [35].

Concerning knowledge of the preventive measures, the highest percentage was reported for keeping surrounding area free from the accumulation of rubbish or waste materials in the present study (81.3%). Similar percentages were observed on keeping good personal hygiene and clean water consumption (77.7% for each). Among communities in The Philippines, 98.0% of their participants knew maintaining good sanitation is important for the prevention [44]. Another communities in Catbalogan City, The Philippines also understood that maintaining the house clean (99.0%) and avoiding the stream water for drinking (73.0%) are also good preventive measures [46]. Rahim et al. [41] reported leptospirosis could be prevented by maintaining the cleanliness of house compound (92.1%). In the present study, avoiding the contaminated fresh water bodies (75.5%) and flood (67.1%) were understood as good preventive measures. Almost similar findings were observed among community residents in The Philippines, i.e., 78.0% and 69.0% of their study participants knew avoidance of going to rivers/streams after the flood and swimming in contaminated water with rat urine, respectively are good preventive practices [44]. A good understanding of appropriate precautionary measures is pivotal in alleviating the public from getting leptospirosis. This is clearly explained by many established reports. For instance, involvement in flood and its related aquatic activities are known to be risk factors for the transmission of leptospirosis [17]. Poor house hygiene and environmental sanitation were also responsible for the increased risk of leptospirosis due to the close proximity to uncollected trash and rodent infestations [8,52].

### 4.2. Attitudes and Preventive Practices on Leptospirosis

In general, the rural communities had unacceptable attitudes in the present study (90.3%). This is reflected by their unacceptable preventive practices (69.1%). Similar data were reported in other studies elsewhere [40,44]. On another note, good knowledge was not reflected by acceptable attitudes and practices for some variables in the present study. For instance, 46.2% and 49.3% of the study participants had strongly agreed to involve their family members in cleaning up their surrounding area and had always cleaned up their housing area, respectively, despite having a good understanding of its cleanliness for prevention (81.3%). Adequate knowledge was also not reflected in their preventive practices as only 39.4% of the participants in the present study had always ensured that there would be no rats around their house. Interestingly, a study in The Philippines demonstrated that despite almost 100% of respondents who had correct knowledge of the preventive measures, only 40.0% of their study participants had really practiced cleaning of their surrounding area [44]. In terms of personal protective equipment (PPE), 37.8% of the participants in the present study had strongly agreed on using gloves, however, only 26.4% of the participants had always used gloves in their actual practice. Our findings corroborate with other reports. Despite having correct knowledge of wearing boots, only 21.0% of the Filipinos used them [44]. There are few factors that have been hypothesized for these findings. For instance, lack of access to PPE was responsible for the low adoption of risk reduction methods by residents in Brazil [35]. Inadequate support for the daily garbage removal by local authorities might also influence the practice level in Brazil [35]. Thus, our rural communities might have encountered the same problems although no data is available to support our notions. Poor health seeking behaviors were also observed in the present study. Only 48.9% of rural communities had strongly agreed to see a doctor if they have fever, while only 40.8% of the participants had gone to see a doctor when they had fever during the leptospirosis outbreak. Commencing appropriate behavior change interventions would be a challenge to respective agencies if these parameters are not carefully evaluated. World Health Organization has recommended that targeting at the source of infection, the route of transmission and health education are important tools for effective prevention and control measures of leptospirosis [53].

### 4.3. Associations between KAP of Leptospirosis with Sociodemographic Factors

Malays were found to have significantly more knowledgeable and acceptable attitudes than non-Malays in the present study. Malays were 2.6 and 2.5 times more likely to have good knowledge and acceptable attitudes, respectively than their counterpart. This could be related to the high incidence of leptospirosis and outbreaks reported among Malays as compared to other ethnicities for a decade [54]. This could trigger them to appropriately response to the disease and be more aware as one of active coping strategies. Health warnings could increase people’s sensitivity to their susceptibility toward diseases [55]. However, no significant association was observed between black and other races of urban slum dwellers in Brazil [35]. Income and education type were significantly associated with attitudes in the present study. Members of rural community who received a formal education were 3.7 times more likely to have good knowledge than those who had non-formal education. This finding re-emphasizes the importance of education as individuals with higher education are able to collect, process and interpret information on healthy behaviors [56]. Lower educational level has been shown to be associated with higher risk of acquiring leptospirosis [57]. Thus, it is hoped that the delivery of health information should be accustomed to our rural communities with a low level of education in accessible and easy-to-understand formats. In addition, many flood victims (mainly rural folks) are gathered in temporary shelters where food and basic amenities are provided for free by the government in Malaysia. Thus, apart from giving physical and emotional support to these people, health information on infectious diseases can be initiated by the community volunteers to raise their awareness especially on minimizing the infection risks. Interestingly, higher income is related to acceptable preventive practices in the present study. Our finding is in concordance with a study among non-agricultural workers in The Philippines [31]. It is very important to note that socioeconomic backgrounds have been recognized as independent risk factors for leptospirosis among residents who live in poverty [52].

In the present study, those who had acceptable attitudes were 4.4 times likely to have acceptable preventive practices than those who had unacceptable attitudes. Available data on the relationships of knowledge, attitudes, and preventive practices of leptospirosis is still lacking. Nonetheless, differences in the causal associations of these parameters have been documented in several studies among zoonotic diseases. Some studies support the significant associations between knowledge and attitudes and preventive practices [58,59], while others do not yield significant associations [60,61].

### 4.4. Limitations

This study has several limitations which need to be highlighted. Findings of our study could not be broadly generalized to all of the rural communities in Malaysia as it was conducted in several rural districts in a state of Malaysia. Different sociocultural and economic backgrounds may give different results. Nonetheless, our findings could offer an insight on the strategies required to effectively address this deadly neglected disease as the number of deaths is increasing each year locally. In addition, since the self-administered questionnaire was used, the risk of having social desirability is anticipated. Moreover, the behavior changes especially on the attitude could not be evaluated as cross-sectional design was used in this study. The questions used were in a close-ended format which could be interpreted differently by the participants.

## 5. Conclusions

This study has demonstrated that the levels of knowledge, attitudes and preventive practices on leptospirosis among our rural communities are still low. Thus, there is an urgent call for the relevant authorities or stakeholders to develop more approachable health education programs or interventions for this group of people and the public at large. There is still a gap in knowledge of leptospirosis, especially on the awareness of the term leptospirosis, its modes of entry, its clinical manifestations and complications. Of our utmost concern, the preventive practices were poorly portrayed, especially on the use of PPEs. These findings could also provide an insight for various agencies such as health district or state offices, schools, universities as well as veterinary and agricultural departments to strengthen their communication and outreach strategy. Mutual involvement between community leaders or volunteers and health professionals should be encouraged to impart knowledge, to strategize and develop good action plans or preventive modules on leptospirosis. Thus, protective health behaviors can be adopted particularly in leptospirosis-prone areas and high-risk populations.

## Figures and Tables

**Table 1:** The knowledge of leptospirosis by participants in rural areas of Hulu Langat.

Question	Correct *n* (%)	Incorrect *n* (%)	Do Not Know *n* (%)
Rat urine disease is also known as leptospirosis	132 (29.7)	5 (1.1)	307 (69.1)
The disease is caused by a bacterium	260 (58.6)	41 (9.8)	143 (32.2)
Infected animals could also infect man	296 (66.7)	36 (8.1)	112 (25.2)
The disease is diagnosed by blood test	271 (61.0)	28 (6.3)	145 (32.7)
The etiological agent may enter human body via:	-	-	-
*Wound site*	152 (34.2)	34 (7.7)	258 (58.1)
*Eyes*	87 (19.6)	47 (10.6)	310 (69.8)
*Nose*	92 (20.7)	41 (9.2)	311 (70.0)
*Mouth*	142 (32.0)	32 (7.2)	270 (60.8)
*Contaminated food*	268 (60.4)	13 (2.9)	163 (36.7)
*Contaminated water/drinks*	275 (61.9)	9 (2.0)	160 (36.0)
*Shaking hands with infected individuals*	57 (12.8)	113 (25.5)	274 (61.7)
An individual with leptospirosis would have:	-	-	-
*Fever*	302 (68.0)	5 (1.1)	137 (30.9)
*Body ache*	233 (52.5)	17 (3.8)	194 (43.7)
*Yellowing of the eyes*	109 (24.5)	40 (9.0)	295 (66.4)
The disease may cause	-	-	-
*Death*	357 (80.4)	5 (1.1)	82 (18.5)
*Breathing difficulty*	201 (45.3)	21 (4.7)	222 (50.0)
*Kidney failure*	173 (39.0)	24 (5.4)	247 (55.6)
*Liver damage*	164 (36.9)	25 (5.6)	255 (57.4)
Leptospirosis can be prevented by:	-	-	-
*Ensuring your residential area free of rubbish*	361 (81.3)	2 (0.5)	81 (18.2)
*Not wading through flood*	298 (67.1)	21 (4.7)	125 (28.2)
*Practising good personal hygiene*	345 (77.7)	7 (1.6)	92 (20.7)
*Drinking clean water supply*	345 (77.7)	0 (0.0)	99 (22.3)
*Wearing rubber gloves*	245 (55.2)	44 (9.9)	155 (34.9)
*Not swimming in contaminated river/lake or waterfall*	335 (75.5)	9 (2.0)	100 (22.5)

**Table 2:** Attitudes of leptospirosis among participants.

Item	Strongly Disagree *n* (%)	Disagree *n* (%)	Maybe *n* (%)	Agree *n* (%)	Strongly Agree *n* (%)
I will wear gloves if I handle the rubbish	16 (3.6)	28 (6.3)	84 (18.9)	148 (33.3)	168 (37.8)
I will make sure my dustbin is always covered	13 (2.9)	8 (1.8)	70 (15.8)	128 (28.8)	225 (50.7)
I will participate in prevention and control activities offered by the authority	13 (2.9)	2 (0.5)	90 (20.3)	139 (31.3)	200 (45.0)
I will make sure my family is involved in cleaning activities around the house	12 (2.7)	0 (0.0)	72 (16.2)	155 (34.9)	205 (46.2)
I will inform the health authority if I find myself having signs/symptoms of leptospirosis	12 (2.7)	2 (0.5)	100 (22.5)	130 (29.3)	200 (45.0)
I will make sure my family members are aware of contaminated river/lake/waterfall	11 (2.5)	3 (0.7)	105 (23.6)	138 (31.1)	187 (42.1)
I am not worried if I wade through the flood	21 (4.7)	53 (11.9)	152 (34.2)	99 (22.3)	119 (26.8)
I do not mind if there are rats in my house and its surrounding	26 (5.9)	14 (3.2)	84 (18.9)	74 (16.7)	246 (55.4)
I will inform the health authority if a person is suspected of having leptospirosis	18 (4.1)	10 (2.3)	120 (27.0)	134 (30.2)	162 (36.5)
I do not mind if my housing area is dirty	17 (3.8)	12 (2.7)	81 (18.2)	69 (15.5)	265 (59.7)
I need to see a doctor if I am having fever during a leptospirosis outbreak	13 (2.9)	2 (0.5)	88 (19.8)	124 (27.9)	217 (48.9)
I need to wear personal protective equipment (rubber boot, facial mask, and others) when I handle the rubbish	13 (2.9)	19 (4.3)	114 (25.7)	147 (33.1)	151 (34.0)
I am not worried if I do not wear personal protective equipment (PPE) such as gloves, boots while handling the rubbish	27 (6.1)	36 (8.1)	117 (26.4)	83 (18.7)	181 (40.8)

**Table 3:** Preventive practices on leptospirosis among rural communities in Hulu Langat.

Preventive Practices	Never *n* (%)	Sometimes *n* (%)	Most of the Time *n* (%)	All the Time *n* (%)	Not Applicable *n* (%)
I ensured no rats are found in my housing area	19 (4.3)	95 (21.4)	66 (14.9)	175 (39.4)	89 (20.0)
I went for a picnic at places contaminated with leptospirosis (waterfall/river/lake) within the past six months	236 (53.2)	62 (14.0)	5 (1.1)	30 (6.8)	111 (25.0)
I cleaned my housing area from any waste/rubbish	15 (3.4)	44 (9.9)	77 (17.3)	219 (49.3)	89 (20.0)
I handled waste/rubbish despite having wounds/cuts on my hands/legs	131 (29.5)	118 (26.6)	43 (9.7)	61 (13.7)	91 (20.5)
I ate food/consumed water while handling the waste/rubbish	287 (64.6)	40 (9.0)	12 (2.7)	16 (3.6)	89 (20.0)
I washed my hands with a soap after managing waste/rubbish	17 (3.8)	35 (7.9)	22 (5.0)	282 (63.5)	88 (19.8)
I wore the following PPE while handling the waste/rubbish:					
*Rubber gloves*	105 (23.6)	85 (19.1)	43 (9.7)	117 (26.4)	94 (21.2)
*Rubber boots*	149 (33.6)	78 (17.6)	33 (7.4)	73 (16.4)	111 (25.0)
*Long sleeve attires*	97 (21.8)	77 (17.3)	45 (10.1)	117 (26.4)	108 (24.3)
I kept my food in a covered container	14 (3.2)	20 (4.5)	41 (9.2)	284 (64.0)	84 (18.9)
I went to see a doctor when I had fever during a leptospirosis outbreak	49 (11.0)	37 (8.3)	59 (13.3)	181 (40.8)	118 (26.6)
I covered the dustbin to prevent rat infestations	12 (2.7)	27 (6.1)	50 (11.3)	272 (61.3)	83 (18.7)
I washed the can/drink box before drinking	57 (12.8)	38 (8.6)	46 (10.4)	219 (49.3)	84 (18.9)
I washed the utensils before cooking	14 (3.2)	27 (6.1)	45 (10.1)	269 (60.6)	89 (20.0)
I preferred a clean restaurant for meals	9 (2.0)	12 (2.7)	43 (9.7)	297 (66.9)	83 (18.7)
I used a plaster on wounds or small cuts while handling the waste/rubbish	31 (7.0)	61 (13.7)	66 (14.9)	189 (42.6)	97 (21.8)
I waded through floods without PPE	146 (32.9)	59 (13.3)	40 (9.0)	39 (8.8)	160 (36.0)
I used a plaster on wounds or small cuts while wading through floods	70 (15.8)	63 (14.2)	42 (9.5)	103 (23.2)	166 (37.4)
I smoked while handling the waste/rubbish	185 (41.7)	18 (4.1)	15 (3.4)	30 (6.8)	196 (44.1)

**Table 4:** Level of attitude among rural communities in relation to socio-demographic variables.

Variables	Attitude Level		*n* (%)	x^2^	*p*	Prevalence Ratio (CI) ^#^
	Acceptable (%)	Unacceptable (%)				
Gender	-	-	-	-	-	-
Male	132 (59.2)	91 (40.8)	223 (50.2)	2.549	0.110	1.219 (0.955–1.555)
Female	147 (66.5)	74 (33.5)	221 (49.8)	-	-	
Age (in years)	-	-	-	-	-	-
<34	147 (67.1)	72 (32.9)	219 (49.3)	3.399	0.065	1.144 (0.991–1.321)
≥34	132 (58.7)	93 (41.3)	225 (50.7)	-	-	
Ethnicity	-	-	-	-	-	-
Malay	246 (66.7)	123 (33.3)	369 (83.1)	13.714	<0.001 *	1.515 (1.162–1.976)
Non-Malay	33 (44.0)	42 (56.0)	75 (16.9)	-	-	
Income (RM) ^†^	-	-	-	-	-	-
<1500	161 (58.8)	113 (41.2)	274 (61.7)	5.098	0.024 *	1.348 (1.032–1.761)
≥1500	118 (69.4)	52 (30.6)	170 (38.3)	-	-	
Education type	-	-	-	-	-	-
Formal	274 (64.0)	154 (36.0)	428 (96.4)	7.092	0.018 *	1.911 (1.341–2.721)
Non-formal	5 (31.3)	11 (68.7)	16 (3.6)	-	-	

* Significant at *p* < 0.05; ^†^ Malaysian Ringgit; ^#^ CI = confidence interval.

**Table 5:** Multiple logistic regression predicting the attitude on leptospirosis.

Variable	β	*p-*Value	Adjusted Odds Ratio	95% CI
Constant		0.001	2.174	-
Ethnicity				
Malay		<0.001 *	0.399	0.239–0.665
Non-Malay	−0.919			
Income (RM) ^†^				
<1500				
≥1500	0.445	0.032 *	1.575	1.041–2.385
Education type				
Non-Formal				
Formal	1.305	0.019 *	3.686	1.237–10.986

Notes: Method = Enter; R^2^ = 72.0%; Overall percentage = 66.0%; * significant *p* < 0.05; ^†^ Malaysian Ringgit.

## Supplementary Materials

The following are available online at http://www.mdpi.com/1660-4601/15/4/693/s1, Table S1: Socio-demographic characteristics of members of a rural community in this study; Table S2: Sources from which members of a rural community obtained the information on leptospirosis; Table S3: Knowledge level of leptospirosis in relation to socio-demographic variables; Table S4: Practices of rural communities relating to prevention of leptospirosis and its socio-demographic variables; Table S5: Multiple logistic regression predicting the knowledge level on leptospirosis; Table S6: Multiple logistic regression predicting the practices level on leptospirosis; Table S7: Association between preventive practices level and knowledge level and attitude of the rural communities related to leptospirosis; Table S8: Multiple logistic regression of knowledge and attitude predicting the practices level on leptospirosis.
