# Supplementary material for: Low Levels of Knowledge, Attitudes and Preventive Practices on Leptospirosis among a Rural Community in Hulu Langat District, Selangor, Malaysia
Source: Int J Environ Res Public Health. 2018 Apr 6;15(4):693. doi: 10.3390/ijerph15040693 (PMC5923735; doi:10.3390/ijerph15040693)
Supplement: Supplementary file 1 [file ijerph-15-00693-s001.pdf]

Supplementary Materials: Low Levels of Knowledge, Attitudes and Preventive Practices on Leptospirosis among a Rural Community in Hulu Langat District, Selangor, Malaysia

Noramira Nozmi, Suhailah Samsudin, Surianti Sukeri, Mohd Nazri Shafei, Wan Mohd Zahiruddin Wan Mohd, Zawaha Idris, Wan Nor Arifin, Norazlin Idris, Siti Nor Sakinah Saudi, Nurul Munirah Abdullah, Zainudin Abdul Wahab, Tengku Zetty Maztura Tengku Jamaluddin, Hejar Abd Rahman, Siti Norbaya Masri, Aziah Daud, Malina Osman and Rukman Awang Hamat

Table S1. Socio-demographic characteristics of members of a rural community in this study.

Variables	*n*	%
Age (in years)	-	-
≥34	219	49.3
<34	225	50.7
Gender	-	-
Male	223	50.2
Female	221	49.8
Ethnicity	-	-
Malay	369	83.1
Chinese	13	2.9
Indian	30	6.8
Others	32	7.2
Marital Status	-	-
Married	290	65.3
Unmarried	154	34.7
Number of Children	-	-
No children	30	10.3
1	46	15.9
2	69	23.8
3	58	20.0
4	47	16.2
5	20	6.9
6	11	3.8
7	4	1.4
8	4	1.4
9	1	0.3
Type of Education	-	-
Formal education	430	96.8
Non-formal education	14	3.2
Working Status	-	-
Employed	278	62.6
Unemployed	166	37.4
Monthly Income (Malaysian Ringgit)	-	-
<RM1500	121	43.5
≥RM1500	157	56.5

Table S2. Sources from which members of a rural community obtained the information on leptospirosis.

Item	Frequency	%
Have heard of rat-urine disease	388	87.4
Television/Radio	293	75.5
Newspaper	198	51.0
Doctor	69	17.8
Poster/Pamphlet	61	15.7
Magazine	60	15.5
Others	21	5.4

Table S3. Knowledge level of leptospirosis in relation to socio-demographic variables.

**Variables**	**Knowledge Level**		***n* (%)**	**x^2**	***p***	**Prevalence Ratio (CI)** #
	**Good (%)**	**Poor (%)**				
Gender	-	-	-	-	-	-
Male	91 (40.8)	132 (59.2)	223 (50.2)	0.893	0.345	1.081 (0.919–1.271)
Female	100 (45.3)	121 (54.7)	221 (49.8)	-	-	
Age (in years)	-	-	-	-	-	-
<34	99 (45.2)	120 (54.8)	219 (49.3)	0.844	0.844	1.106 (0.892–1.370)
≥34	92 (40.9)	133 (59.1)	225 (50.7)	-	-	
Ethnicity	-	-	-	-	-	-
Malay	172 (46.6)	197 (53.4)	369 (83.1)	11.514	0.001 *	1.840 (1.229–2.755)
Non-Malay	19 (25.3)	56 (74.7)	75 (16.9)	-	-	
Income (RM) †	-	-	-	-	-	-
<1500	113 (41.2)	161 (58.8)	274 (61.7)	0.922	0.337	1.086 (0.916–1.287)
≥1500	78 (45.9)	92 (54.1)	170 (38.3)	-	-	
Education type	-	-	-	-	-	-
Formal	184 (42.9)	244 (57.1)	428 (96.4)	0.004	0.952	0.987 (0.636–1.532)
Non-formal	7 (43.7)	9 (56.3)	16 (3.6)	-	-	

* Significant at $p < 0.05$; † Malaysian Ringgit; # CI = confidence interval.

Table S4. Practices of rural communities relating to prevention of leptospirosis and its socio-demographic variables.

**Variables**	**Practices Level**		***n* (%)**	**x^2**	***p***	**Prevalence Ratio (CI)** #
	**Good (%)**	**Unacceptable (%)**				
Gender	-	-	-	-	-	-
Male	67 (30)	156 (70)	223 (50.2)	0.138	0.710	1.024 (0.904–1.159)
Female	70 (31.7)	151 (68.3)	221 (49.8)	-	-	
Age (in years)	-	-	-	-	-	-
<34	69 (31.5)	150 (68.5)	219 (49.3)	0.086	0.770	1.043 (0.789–1.377)
≥34	68 (30.2)	157 (69.8)	225 (50.7)	-	-	
Ethnicity	-	-	-	-	-	-
Malay	114 (30.9)	255 (69.1)	369 (83.1)	0.002	0.969	1.007 (0.694–1.463)
Non-Malay	23 (30.7)	52 (69.3)	75 (16.9)	-	-	
Income (RM) †	-	-	-	-	-	-
<1500	71 (25.9)	203 (74.1)	274 (61.7)	8.197	0.004 *	1.211 (1.054–1.391)
≥1500	66 (38.8)	104 (61.2)	170 (38.3)	-	-	
Education type	-	-	-	-	-	-
Formal	132 (30.8)	296 (69.2)	428 (96.4)	0.001	1.000	0.994 (0.710–1.392)
Non-formal	5 (31.3)	11 (68.7)	16 (3.6)	-	-	

* Significant at $p < 0.05$; † Malaysian Ringgit; # CI = confidence interval.

Table S5. Multiple logistic regression predicting the knowledge level on leptospirosis.

Variable	β	*p* Value	Adjusted Odds Ratio	95% CI
Constant		0.017	2.247	-
Ethnicity				
Malay<br>Non-Malay	−0.945	0.001 *	0.389	0.222–0.680

Notes: Method = Enter; R^2 = 36.0%; Overall percentage = 57.0%; (*)—Significant $p < 0.05$.

Table S6. Multiple logistic regression predicting the practices level on leptospirosis.

Variable	β	*p* Value	Adjusted Odds Ratio	95% CI
Constant		<0.001	0.193	-
Income (RM) †				
<1500<br>≥1500	0.596	0.004 *	1.814	1.204–2.734

Notes: Method = Enter; R^2 = 25.0%; Overall percentage = 69.1%; (*)—Significant $p < 0.05$; † Malaysian Ringgit.

Table S7. Association between preventive practices level and knowledge level and attitude of the rural communities related to leptospirosis.

Variables	Practices Level (%)		*n* (%)	x^2	*p*	Prevalence Ratio (CI) #
	Good (%)	Unacceptable (%)				
Knowledge Level						
Good	71 (37.2)	120 (62.8)	191 (43.0)	6.269	0.012 *	0.702 (0.532–0.926)
Poor	66 (26.1)	187 (73.9)	253 (57.0)			
Attitude Level						
Acceptable	115 (41.2)	164 (58.8)	279 (62.8)	37.789	<0.001 *	0.323 (0.214–0.489)
Unacceptable	22 (13.3)	143 (86.7)	165 (37.2)			

* Significant at $p < 0.05$; # CI = confidence interval.

Table S8. Multiple logistic regression of knowledge and attitude predicting the practices level on leptospirosis.

Variable	β	*p* Value	Adjusted Odds Ratio	95% CI
Constant		<0.001	0.021	-
Knowledge Level				
Good<br>Poor	0.371	0.087	1.449	0.948–2.215
Attitude Level				
Acceptable<br>Unacceptable	1.472	<0.001 *	4.357	2.613–7.264

Notes: Method = Enter; R^2 = 13.3%; Overall percentage = 69.1%; (*)—Significant $p < 0.05$.

© 2018 by the authors; licensee MDPI, Basel, Switzerland. This article is an open access article distributed under the terms and conditions of the Creative Commons by Attribution (CC-BY) license (http://creativecommons.org/licenses/by/4.0/).
